# Reusable SERS Substrates Based on Gold Nanoparticles for Peptide Detection

**DOI:** 10.3390/s23146352

**Published:** 2023-07-13

**Authors:** Zhang Qi, Timur Akhmetzhanov, Arina Pavlova, Evgeny Smirnov

**Affiliations:** 1 Department of Material Science, Shenzhen MSU-BIT University, International University Park Road 1, Dayun New Town, Longgang District, Shenzhen 518172, China; 2 Infochemistry Scientific Center, ITMO University, Lomonosova Str. 9, Saint Petersburg 191002, Russia

**Keywords:** biosensor, bovine serum albumin, core–shell nanoparticles, enhancing substrates, gold nanoparticles, malachite green, surface-enhanced Raman spectroscopy

## Abstract

Raman spectroscopy is a powerful analytical technique widely used for quantitative and qualitative analysis. However, the development of inexpensive, reproducible, and reusable enhancing substrates remains a challenge for material scientists and analytical chemists. In this study, we address this challenge by demonstrating the deposition of core–shell nanoparticles consisting of a gold core and a thin inert SiO_2_ shell within a confined space, resulting in the formation of a highly efficient Raman-enhancing structure. Nanoparticles were characterized by UV–vis spectroscopy, dynamic light scattering, and total reflectance X-ray fluorescence spectroscopy, whereas the prepared substrates were characterized by scanning electron microscopy and Raman spectroscopy with a model molecule, malachite green. The relationship between Raman intensity and the loading of malachite green dye exhibited linearity, indicating the uniform spatial distribution of hotspots across the substrate. The limit of detection was determined as 2.9 μM of malachite green when 10 uL was distributed over a ca. 25 mm^2^ surface area. Moreover, the same substrate, after thorough washing in ethanol, was successfully employed for the detection of bovine serum albumin at a concentration level of 55 μg mL^−1^, demonstrating its reusability and versatility. Our findings highlight the potential of these substrates for various applications in biomedical research, clinical diagnosis, and beyond.

## 1. Introduction

Over the past century, Raman spectroscopy has developed into a powerful analytical tool in various fields, including chemistry [1], biology [2,3], ecology [4,5], medicine [6], and even art [7]. The discovery of surface-enhanced Raman spectroscopy (SERS) on the rough surface of a silver electrode by Van Duyne in 1977 [8] played a significant role in the development of Raman spectroscopy as an analytical method, as the main limitation of Raman spectroscopy—the low intensity of the scattering—had been overcome. The sensitivity of SERS is highly dependent on the properties of the substrate used for signal enhancement [9]. The need to develop an inexpensive, reproducible, and reusable substrate was highlighted by Marzan with colleagues in Ref. [9] as one of the top 10 challenges in the field of Raman spectroscopy. Therefore, the development of efficient and reliable SERS substrates is of great interest to researchers.

Today, the origin of Raman enhancement is described with electromagnetic (EMM) and chemical (ChM) mechanisms. The electromagnetic mechanism is associated with plasmon excitation in metallic particles rich with electrons, as well as plasmon interaction in close proximity to the particle surface. The enhancement factor (EF) for the electromagnetic mechanism is typically about 10^5^–10^6^ in comparison with regular Raman measurements, whereas the chemical mechanism adds another order or two to the EF due to charge transfer between either the molecules themselves [10] or the absorbed molecule and the substrate [11,12]. Thus, the enhancement factor could reach 10^6^–10^8^. 

In recent years, gold nanoparticles (AuNPs) have emerged as one of the most promising materials for SERS substrates due to their unique chemical (relatively stable to oxidation and surface reactions), optical (matching with common laser wavelength used in SERS), and electronic properties (electron “storage” for chemical enhancement). A number of methods to synthesize AuNPs with different sizes and shapes have been developed, although the classical approach known as Frens–Turkevich synthesis [13] prevails, along with further seed-mediated growth [14,15] as an inexpensive, high throughput, and accessible preparation method.

The advances in nanotechnology have enabled the development of Raman-enhancing particles covered with a thin layer of oxides such as silica or alumina, the so-called shell-isolated nanoparticle-enhanced Raman spectroscopy (SHINERS). SHINERS have greatly enhanced the sensitivity of Raman spectroscopy and allowed for the detection of trace amounts of molecules, making them useful in a variety of fields, such as infectious disease diagnostics and food safety monitoring [16]. Despite the chemical inertness of gold nanoparticles, the nanoparticles may still react with molecules present in the analyte solution, not only charging or discharging the nanoparticles [17,18] but also changing the conformation of complex molecules, for example, peptides, due to the specific affinity of gold to amino and thiol groups. The presence of a thin oxide shell around the gold core minimizes these interactions and provides a platform for surface modification to be implemented in biological and medical studies, which require improved biocompatibility [19,20]. Nonetheless, to achieve the best results in Raman spectroscopy, such an oxide shell should be as thin as possible, as the intensity of Raman scattering drastically decreases with the thickness of the shell. Typical values of the shell should not exceed 4 to 5 nm, according to Ref. [21].

Raman spectroscopy has been widely reported and recognized for its capability to detect a diverse range of small peptide molecules such as bovine serum albumin (BSA) [22,23]. Moreover, the versatility of Raman spectroscopy extends beyond the characterization of individual molecules, as it has demonstrated the remarkable ability to investigate complex biological systems, including the detection and analysis of whole living cells [2,3]. By providing detailed molecular information about cellular components and their interactions through the fingerprint region [2] and DFT-modeling [23] of Raman spectra of complex molecules, Raman spectroscopy has emerged as a valuable tool in cellular biology and biomedical research. The latter enables the route to non-destructive, label-free, and real-time analysis of cellular processes and dynamics. In this scenario, assemblies of plasmonic gold core–silica shell nanoparticles are unique and suitable candidates as enhancing substrates for SERS using red lasers (633 and 785 nm).

Finally, for successful analytical application, it is crucial that Raman-enhancing substrates exhibit not only enhanced signals but also maintain uniformity and linearity across a wide range of analyte concentrations [9]. The reliability and performance of SERS substrates must be thoroughly assessed before practical implementation, and this can be achieved through comprehensive testing using well-established model molecules. In this regard, various dyes such as rhodamine 6 G [24] and/or malachite green [25], which are commonly employed as SERS probe molecules, serve as valuable indicators to evaluate the substrate’s effectiveness and suitability for subsequent analytical tasks. Malachite green is frequently employed as a dye in various applications, including environmental monitoring [26] and food safety [27].

In this study, we present a novel approach for the preparation of reusable SERS substrates based on gold nanoparticles with a silica shell deposited on a glass slide within a confined space. The present configuration was designed to minimize the undesirable “coffee-ring” effect commonly observed in conventional deposition methods, ensuring uniformity and reproducibility in the deposition process, which is crucial for the practical applications of SERS substrates. Such uniformity and reproducibility in the deposition process will directly affect the quality of the substrate and its enhancing properties. The synthesized nanoparticles were thoroughly characterized by instrumental methods. The enhancing properties of the prepared SERS substrates were investigated using Raman spectroscopy, employing malachite green as a model molecule. Furthermore, the substrates demonstrated excellent stability and reproducibility as shown by Raman mapping of the sample surface, allowing for repeated detection and washing of deposited analyte molecules without any visible damage. Finally, in a practical demonstration of their capabilities, we applied the substrates to detect bovine serum albumin (BSA), a widely studied peptide, known for its relevance in biomedical research and clinical diagnosis. The results clearly indicated that our SERS substrates were capable of detecting BSA at low concentrations, revealing their high sensitivity and potential utility in the field of bioanalysis, biomedical research, and clinical diagnosis.

## 2. Materials and Methods

*Reagents.* Solutions of tetrachloroauric acid in HCl (HAuCl_4_, 23.5–23.8 w% Au), (3-aminopropyl)triethoxysilane (APTES, C_9_H_23_NO_3_Si, 98%) were purchased from Aladdin, China. Sodium citrate (Na_3_C_6_H_5_O_7_·2H_2_O, 98%) and silver nitrate (AgNO_3_, 99.9%) were received from Innochem, China. Ascorbic acid (99.9%) was bought from Meligen, Russia. Sodium silicate (Na_2_SiO_3_·9H_2_O) was ordered from Shanghai Lingfeng Chemical Reagent Co., Ltd., China. Malachite green (MG, C_23_H_25_N_2_Cl, 96%) and concentrated HCl were received from Macklin, China. Bovine serum albumin (BSA, 96%) was bought from Sigma-Aldrich, Burlington, Massachusetts, USA.

All chemicals were used as received without further purification. In all experiments, MilliQ water (18.2 MΩ cm) was used.

*Synthesis of gold nanoparticles.* Nanoparticles were synthesized according to the previously published method [28,29] with the modifications considered in Ref. [15]. Briefly, 47.5 mg of the solution containing 23.5 w% of gold was added to 100 mL of MiliQ water and then boiled in a round-bottom flask. Then, 7.5 mL of 1 w% solution of sodium citrate was rapidly injected into the flask. In 30 min, the solution turned from pale yellow to dark red, leading to the formation of so-called “seed nanoparticles”.

Further, seed nanoparticles were grown according to the procedure described in [14] via a seed-mediated growth method. In a nutshell, 75 mg HAuCl_4_ solution and 0.4 mL 10 mM AgNO_3_ were added in a round-bottom flask with 170 mL of deionized water with the subsequent addition of 15 mL of seed nanoparticles. After that, 30 mL of 5 mM solution of ascorbic acid was added dropwise at the rate of 0.5 mL min^−1^.

*Synthesis of core–shell particles.* To cover gold nanoparticles with a thin shell of silica, the APTES-mediated procedure was applied [20,21]. In a beaker, 30 mL of gold nanoparticles was mixed with 0.4 mL of 1 mM APTES solution and left for 15 min to complete a reaction upon vigorous stirring. Then, 3.2 mL of 0.5 w% sodium silicate solution with pH = 10.8 was added upon stirring. After 3 min of continuous stirring, the beaker was moved to an oil bath conditioned at 90 °C and left for 1.5 h to complete the reaction. 

*Preparation of enhancing substrate.* The substrates were prepared on glass slides that were thoroughly cleaned with oxygen plasma prior to the deposition process. To create a confined space with a consistent deposition surface area of approximately 25 mm^2^ and mitigate the coffee-ring effect to some extent, the drop-casting method was employed. Specifically, 5 to 30 μL of the gold nanoparticle solution was carefully deposited within the confined space delimited by Kapton tape.

**Scheme 1:**
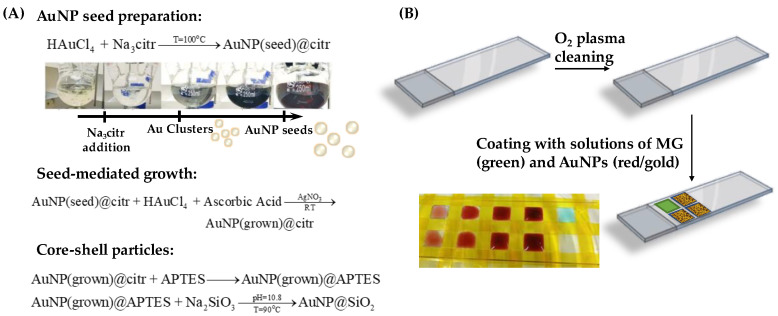
Schematic illustration of (**A**) the gold nanoparticle (AuNP) synthesis and the core–shell preparation, (**B**) preparation of the enhancing substrates involving drop-casting the AuNP solution onto cleaned glass slides in a space confined by Kapton tape.

As illustrated in Scheme 1, it can be observed that deposition volumes of 5, 10, and 20 μL were insufficient to cover the entire designated surface area adequately. Therefore, for all experimental procedures, a deposition volume of 30 μL was utilized to ensure complete coverage and uniformity across the substrate.

To clean the enhancing substrates and remove any residual dye or peptide, a thorough washing process was carried out using an ethanol solution. The substrates were subjected to multiple washing cycles to ensure efficient removal of the analyte molecules. This cleaning step was essential to maintain the substrate’s reusability and ensure that subsequent measurements would not be influenced by previous analyte deposits.

*UV–vis spectroscopy.* Absorption spectra in the UV–visible regions were recorded using a high-quality spectrophotometer T9 (PGENERAL, Beijing, China) with a wavelength range of 350 to 800 nm and a precision of 1 nm. The mean diameter and concentration of the particles were determined based on previously published results [15,30].

*Dynamic light scattering (DLS) and zeta potential.* Dynamic light scattering (DLS) and zeta potential measurements were performed using a Litesizer 500 instrument (Anton Paar, Austria). The instrument utilized laser irradiation at a wavelength of λ = 658 nm. DLS measurements were carried out in a glass cell, whereas a specially designed cell with incorporated electrodes were used for zeta potential measurements.

*Scanning electron microscopy (SEM).* SEM images were acquired using an EM-6200 microscope (KYKY, China) operating at an acceleration voltage of 20 keV.

*Transmission electron microscopy (TEM).* TEM images were acquired using a Tecnai Spirit microscope (FEI, Netherlands) operating at an acceleration voltage of 120 keV.

*Total reflection X-ray fluorescence (TXRF).* The composition of the samples was analyzed qualitatively and quantitatively using total reflection X-ray fluorescence (TXRF) with an XRF S4 T-STAR instrument from Bruker (Germany). To perform the quantitative analysis, a nanoparticle sample volume of 100 μL was mixed with a predetermined quantity of GaCl_3_ in an HCl solution. Subsequently, the mixture was deposited onto an acrylic substrate and dried at a temperature of 50 °C for a duration of 15 min. To ensure the accuracy and reliability of the measurements, each sample underwent 3 to 5 independent measurements.

*Raman spectra measurements.* Raman spectra were acquired using a Raman microscope InVia (Renishaw, Wotton-under-Edge, UK) equipped with a 633 nm laser. The laser delivered a power of approximately 0.1 mW on the sample surface. The typical time to acquire the SERS spectrum was 5 s with 5 accumulations. All data were processed using the WiRE software, which provided tools for data analysis and spectral interpretation.

The typical procedure for measuring Raman spectra involved the deposition of 10 μL of malachite green (MG) dye solution on the surface. The dye concentrations ranged from 10^−7^ M to 10^−5^ M in ethanol to intensify the drying process. After the droplet had dried completely, Raman spectra were recorded. To calculate the enhancement factor (EF), the Raman spectrum of pristine MG was recorded after being deposited on a pristine glass surface of 30 μL of a 10^−4^ M MG solution. In the case of bovine serum albumin (BSA), a similar procedure was followed, but the ethanol was replaced with an aqueous solution.

To ensure statistical significance and accuracy, 3 to 5 separate points on the surface were measured for each concentration of MG. This allowed for the calculation of error bars, providing a measure of the variability within the measurements and ensuring reliable data interpretation and analysis.

For mapping experiments, a grid of 100 × 100 points was scanned with a step size of 2 μm between each point. This allowed for the acquisition of spatially resolved Raman data, enabling the investigation of sample heterogeneity and the identification of localized SERS hotspots. All maps were treated and plotted with WiRE software (version 5.3).

## 3. Results and Discussion

### 3.1. Characterization of Synthesized Gold Nanoparticles

**Figure 1:**
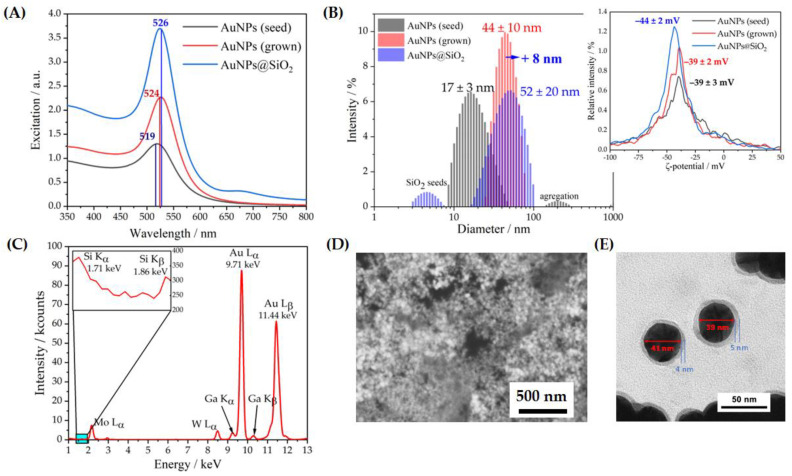
Characterization of synthesized gold nanoparticles. (**A**) UV–vis spectra for seed particles, nanoparticles after seed-mediated growth, and core–shell particles. (**B**) DLS mean particle size distribution. Insert: zeta potential distribution of synthesized particles. (**C**) TXRF data used to calculate the mean SiO_2_ shell thickness. (**D**) SEM and (**E**) images used to determine the particle diameter of core–shell nanoparticles and core thickness.

Gold nanoparticles were synthesized following the procedure described earlier. The characterization of the gold nanoparticles is presented in Figure 1.

In Figure 1A, the absorbance spectra of the seed gold nanoparticles reveal a peak at 519 nm, indicating a mean diameter of 13 nm. With the process of seed-mediated growth, a red shift in the absorbance peak is observed, reaching 524 nm, corresponding to an enlargement of the particles to 28 nm. Subsequently, the growth of the SiO_2_ shell on the gold core causes a further 2 nm red shift. This shift can be attributed to changes in the dielectric constant as well as an overall increase in the diameter of the nanoparticles.

To determine the concentrations of gold nanoparticles in the solution, Haiss’s approach [30] was utilized. The latter shows a dramatic drop in concentration between the initial seed particles and the AuNPs obtained after the seed-mediated growth process, primarily due to dilution effects. However, after the deposition of the silica shell, the concentration of the nanoparticles remains relatively unchanged. This observation can be attributed to the alteration in the dielectric constant of the shell, as the citrate–water medium is replaced by silica. The summarized results of these characterizations are provided in Table 1.

Similarly, DLS and zeta potential measurements were performed to evaluate the changes in the particle diameter and surface charge during the synthesis process (Figure 1B). The results are shown in Table 1. Thus, DLS analysis revealed a significant increase in the mean particle diameter from 17 nm to 44 nm following the seed-mediated growth process. However, the subsequent addition of the silica shell resulted in a comparatively smaller increase of only 8 nm in the mean diameter, indicating a controlled growth process.

Furthermore, zeta potential measurements provided insights into the surface charge of the nanoparticles. Initially, both types of gold nanoparticles exhibited a zeta potential of approximately −39 mV. The addition of a silica shell shifted the zeta potential to −43 mV. This shift toward a more negative value can be attributed to the presence of the negatively charged SiO_2_ surface, which contributes to the overall surface charge of the nanoparticles.

To validate the mean particle diameter obtained from Table 1, SEM images were acquired, and the diameter of several particles was measured, yielding an average of 50 ± 5 nm. This measurement aligns well with the data presented in Table 1, confirming the accuracy and reliability of the particle size characterization.

Further, to confirm the presence of silica in the synthesized samples, total reflection X-ray fluorescence (TXRF) analysis was performed on acrylic sample holders (Figure 1C). In addition to the characteristic L_α_ and L_β_ lines of gold observed at 9.71 and 11.44 keV, respectively, and the Ga lines used as an internal standard, the TXRF spectrum also revealed the presence of small peaks corresponding to the Si K_α_ and K_β_ lines at 1.71 and 1.86 keV, respectively [31].

Notably, to remove any excess unreacted silica and large silica aggregates, the samples underwent multiple washes with deionized water followed by centrifugation and redispersion. This rigorous purification process ensures that only the desired silica shell remains on the gold core and very small SiO_2_ particles. By using TXRF, it becomes possible to estimate the maximum thickness of the silica shell on the gold core by calculating the concentration ratio between gold and silicon (Au/Si). To that end, the concentration ratio between Au and Si should be calculated as follows:

(1) $$\frac{C_{\mathrm{Au}}}{C_{\mathrm{Si}}}=\frac{V_{\mathrm{Au}}\cdot \rho_{\mathrm{Au}}}{V_{{\mathrm{SiO}}_{2}}\cdot \rho_{{\mathrm{SiO}}_{2}}\cdot \omega_{{\mathrm{Si}\;\mathrm{in}\;\mathrm{SiO}}_{2}}},$$

where *C*_i_—element concentration from the TXRF experiment in mg L^−1^; *V*_i_—volume; *ρ*_i_—crystallographic density in g cm^–3^; and *ω*—mass % of Si in SiO_2_. *V*_Au_ and *V*_SiO2_ can be calculated from geometry as follows:

(2) $$V_{\mathrm{Au}}=\frac{4}{3}\pi r^{3},$$

(3) $$V_{{\mathrm{SiO}}_{2}}=\frac{4}{3}\pi ({\left(r+h\right)}^{3}-r^{3}),$$

where *r*—radius of the gold core and *h*—thickness of the silica shell.

Therefore, taking into account Equations (1)–(3) along with measured *C*_Si_ = 511 mg L^−1^ and *C*_Au_ = 2561 mg L^−1^, the thickness (*h*) can be estimated as 9.5 nm. Additionally, the thickness of the SiO_2_ shell was estimated using the mentioned above UV–vis spectra and DLS particle size distributions. The shell thickness was calculated as half of the difference between the diameters of particles with and without the shell. The UV–vis analysis yielded an estimated shell thickness of 3.5 nm. On the other hand, DLS measurements provided a slightly different estimation, with a shell thickness of 4 ± 5 nm. According to TEM, shell thickness can be estimated as 4 to 5 nm (Figure 1E). Overall, all the mentioned methods indicate that the SiO_2_ shell is relatively thin, which is in line with the intended design of the enhancing substrates.

Therefore, the silica shell deposited on the surface of the gold nanoparticles is relatively thin, which minimally interferes with Raman measurements. However, its presence serves as a protective barrier, shielding the gold surface from contamination and preventing unexpected reactions with analytes.

### 3.2. Characterization of the Obtained SERS Substrates

**Figure 2:**
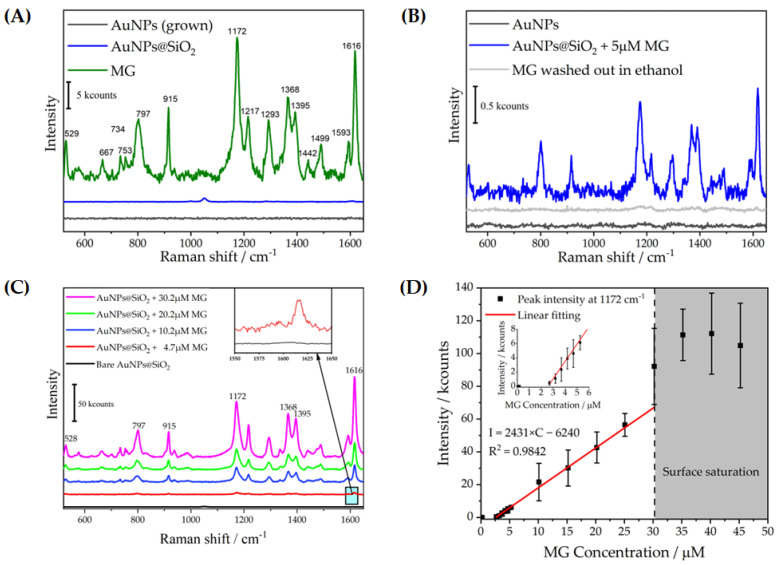
Characterization of SERS substrates with malachite green dye. (**A**) Comparison of Raman spectra for all synthesized gold nanoparticles and 30 μL of 100 μM malachite green dye (MG). (**B**) Comparison of Raman spectra using the SERS regime for covered and non-covered particles with silica shell with 5 μM of malachite green dye. (**C**) Raman spectra at increasing MG concentrations from 0.1 μM to 30.2 μM of malachite green dye. (**D**) Calibration curve demonstrating the linear dependence of Raman scattering of MG at 1172 cm^−1^. Insert: scaled-up low-concentration region.

After the preparation of the enhancing substrates through drop-casting in the confined space, the substrates were further characterized using malachite green (MG) dye deposition. The experimental procedure is described in detail in the Materials and Methods section, Raman spectra measurements. In summary, the MG dye was added drop-wise onto the substrate surface, followed by drying, and the subsequent Raman spectra measurements (Figure 2).

The characterization of the prepared enhancing substrates, consisting of different types of gold nanoparticles with and without a silica shell, is presented in Figure 2. As depicted in Figure 2A, both types of substrates, with and without the silica shell, exhibit minimal background signals compared to the MG spectrum. However, the presence of the thin silica shell, as discussed earlier (approximately 3–4 nm), significantly enhances the substrate’s performance by allowing the MG molecules to access the hotspot regions located between the nanoparticles (Figure 2B). Moreover, MG molecules were easily washed out from the surface after several washing steps in an ethanol solution (Figure 2B, gray line).

This improvement can be attributed to the possibility of pin-holes within the silica shell, as described in Ref. [21], where dye molecules can adsorb and contribute to the enhanced Raman signal. The characterization results presented in Figure 2B demonstrate the efficacy of the silica shell in facilitating the enhancement of Raman signals and the favorable interaction between the dye molecules and the substrate surface.

Two of the most prominent bands corresponding to benzene ring vibrations in the malachite green dye were selected for further experimentation and calculation of enhancement factors. These bands are located at 1172 cm^−1^ (in-plane C-H (ring), δ(C-H)) and 1616 cm^−1^ (stretching of the benzene ring). The observed MG bands and their corresponding vibrational types are summarized in Table 2.

**Figure 3:**
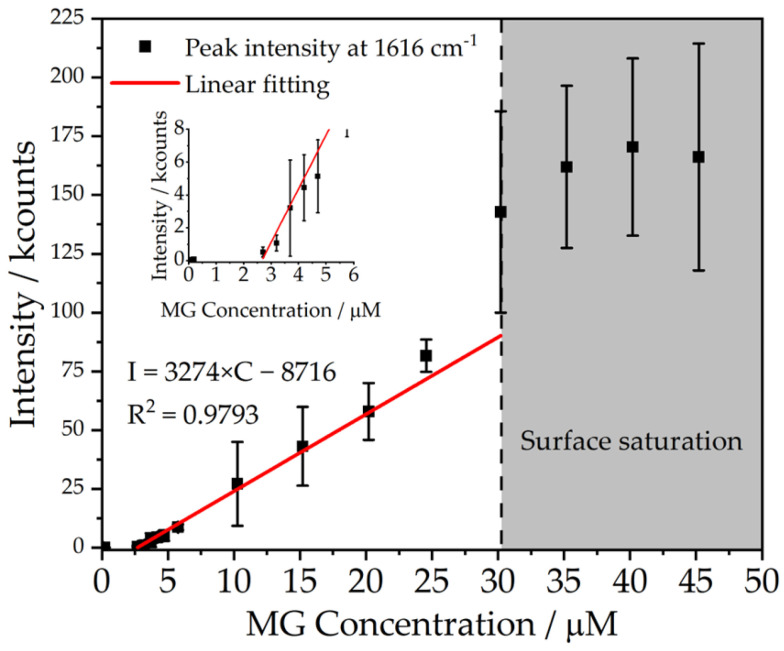
Calibration curve demonstrating the linear dependence of Raman scattering of MG at 1616 cm^−1^. Insert: scaled-up low-concentration region.

To investigate the loading of MG to the substrate surface, a gradual increase in MG concentration was achieved by successive deposition of MG solutions onto the enhancing substrate (Figure 2C). Importantly, all measurements were conducted from the same or nearly the same spot on the substrate surface, due to the fully motorized sample holder, with two to four random spots around. This approach effectively minimized the influence of substrate morphology on the results. The Raman scattering signal exhibited a gradual increase with increasing MG concentrations, indicating a linear dependence with a high R^2^ factor reaching 0.9842 and 0.9793 for the 1172 cm^−1^ and 1616 cm^−1^ bands, respectively. These linear dependencies are illustrated in Figure 2D for the 1172 cm^−1^ band and in Figure 3 for the 1616 cm^−1^ band. At high MG concentrations, the saturation of the surface with MG molecules can be observed, as the scattering intensity reaches a plateau with relatively high intensity and high relative errors.

Furthermore, based on the presented data and observed linear dependence, the limit of detection (LOD) was calculated. The LOD values were determined to be 2.94 μM and 2.89 μM of MG for the 1172 cm^−1^ and 1616 cm^−1^ bands, respectively. 

The linearity of the Raman scattering intensity observed in the previous experiments further confirms the high quality of the enhancing substrate. The sensitivity can be rationalized as a derivative of the intensity over the concentration range. In both cases, the observed coefficient is relatively high (2431 counts μM^−1^ and 3274 counts μM^−1^, respectively).

**Figure 4:**
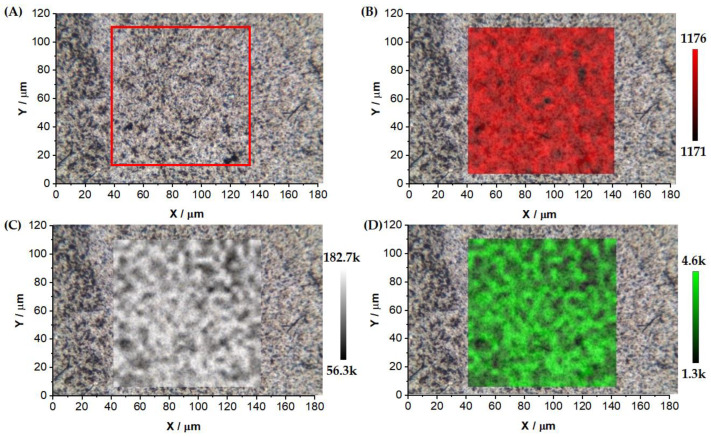
Characterization of SERS-enhancing surfaces with malachite green dye at 1172 cm^−1^. (**A**) Morphology of the substrate at 50× magnification. (**B**) Exact peak position of the band. (**C**) Calculated peak surface area and (**D**) peak intensity maps.

To provide additional evidence of the substrate’s performance, mapping of the enhancing surface was conducted, resulting in a hyperspectral map (Figure 4).

The concentration of MG used for mapping was set at 4.5 μM, which is close to the lower end of the linear dependence range.

Figure 4A illustrates the morphology of the enhancing surface, which appears regular without significant voids and relatively flat. Subsequently, three maps were generated from the hyperspectral map to visualize specific characteristics of the Raman signal. Thus, Figure 4B shows the exact peak position of the selected band (1172 cm^−1^), while Figure 4C represents the surface area associated with the peak. Lastly, Figure 4D displays the peak intensity distribution across the surface. As observed from Figure 4B–D, the enhancing surface exhibits a uniform distribution of hotspots, and these hotspots are attributed to the presence of gold nanoparticles. Although there may be slight variations in the signal intensity, these spatial signal variations are below a few microns. 

**Figure 5:**
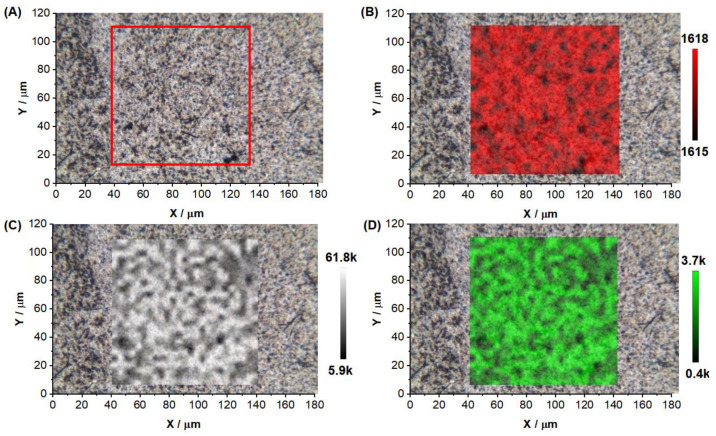
Characterization of SERS-enhancing surfaces with malachite green dye at 1616 cm^−1^. (**A**) Morphology of the substrate at 50× magnification. (**B**) Exact peak position of the band. (**C**) Calculated peak surface area and (**D**) peak intensity maps.

Additional mapping information for the 1616 cm^−1^ band can be found in Figure 5. This map provides insights into the spatial distribution of the peak intensity at 1616 cm^−1^ across the enhancing surface. Similar to the observations made for the 1172 cm^−1^ band, the mapping results for the 1616 cm^−1^ band demonstrate the uniformity and consistent distribution of the hotspots associated with the presence of gold nanoparticles.

To complete the substrate characterization, the enhancement factor of the substrates was calculated to evaluate their performance. The analytical enhancement factor (AEF) was determined using the following calculation method, as described in Refs. [36,37,38]:

(4) $$\mathrm{AEF}=\frac{I_{\mathrm{SERS}}}{C_{\mathrm{SERS}}^{\mathrm{surf}}}\frac{C_{\mathrm{RS}}^{\mathrm{surf}}}{I_{\mathrm{RS}}}$$

where $I_{\mathrm{SERS}}$ and $I_{\mathrm{RS}}$ are the intensities for the given band in enhancing condition and at regular Raman scattering, respectively; $C_{\mathrm{SERS}}^{\mathrm{surf}}$ and $C_{\mathrm{RS}}^{\mathrm{surf}}$ are the surface concentrations of the scattering molecules in the case of SERS and regular Raman regimes.

Taking into account Equation (4), AEF can be calculated as 3.4 × 10^4^ and 5.4 × 10^4^ for the most intensive bands at 1172 and 1616 cm^−1^, correspondingly. 

### 3.3. SERS Analysis of Peptides: A Case of BSA

**Figure 6:**
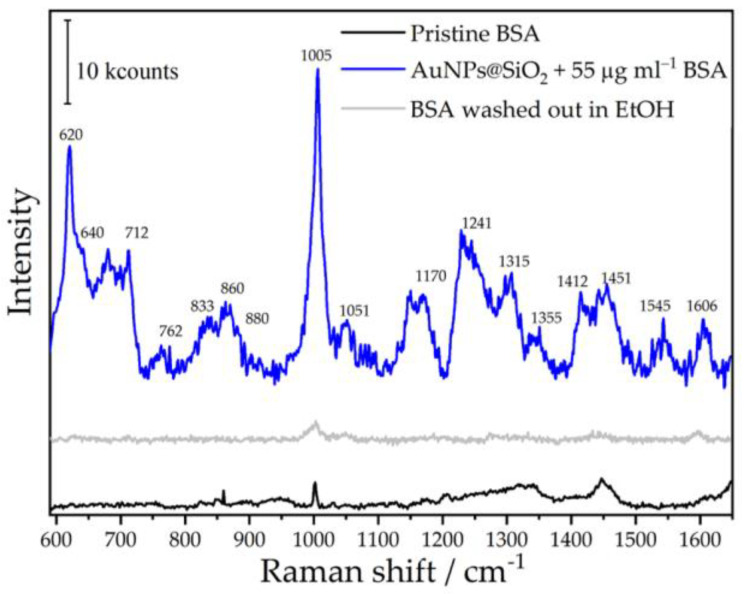
Raman spectra of BSA with and without enhancement on the AuNPs@SiO_2_ core–shell substrate.

The synthesized enhancing substrates, consisting of gold nanoparticles with a thin silica shell, were utilized to detect BSA, serving as a model peptide. Notably, the same substrates were employed for BSA detection following the removal of MG dye through serial washing steps in an ethanol solution. Figure 6 illustrates a comparison between the enhanced and non-enhanced Raman spectra of BSA. In its pristine form, BSA exhibits a low Raman scattering intensity with poorly resolved bands (Figure 6, black line). Only a single band, located at approximately 1005 cm^−1^, can be easily identified. However, the application of the enhancing substrate enables the resolution of all bands in the spectrum and facilitates their assignment to specific amino acids present in BSA (Figure 6, blue line).

The whole Raman spectrum of BSA consisted of amino acid fingerprints in proportion to their presence in the peptide. The Raman spectrum of BSA exhibits several distinctive bands that can be attributed to specific amino acids present in its peptide structure [23]. The highest amount of amino acid residues belongs to Lysine (59 residues in BSA); however, only a few middle-intensity bands are present in the spectrum at 1412 cm^−1^ and 1451 cm^−1^_._ The other most common amino acid is phenylalanine, with 24 residues in BSA. Phenylalanine gives rise to intense bands at 620 cm^−1^, 1005 cm^−1^, and 1606 cm^−1^, which are characteristic of the phenyl ring vibrations, however the band at 1583 — 1587 cm^−1^ was not observed clearly. Another amino acid—Tyrosine, with 20 residues in BSA—shows the fermi doublet at 833 cm^−1^ and 859 cm^−1^, as well as additional bands at 640 cm^−1^ and 1170 cm^−1^. Histidine, with 17 residues, can be observed through bands at 1315 cm^−1^. Tryptophan, with two residues, exhibits bands at 880 cm^−1^, 1350 cm^−1^, and 1545 cm^−1^. Leucine, although difficult to identify due to limited specific bands, shows a band at 1451 cm^−1^ corresponding to stretching in the CH_3_ group. Methionine can be identified by the characteristic C-S vibration at 712 cm^−1^ and stretching in the CH_3_ group at 1451 cm^−1^. The extended band between approximately 1250 cm^−1^ and 1350 cm^−1^ can be attributed to various amide bonds in BSA. Table 3 provides a comprehensive summary of the observed vibrational bands in the Raman spectrum of BSA, along with their corresponding attributions to the amino acids present in the structure of BSA.

These assignments provide valuable insights into the specific vibrational modes of the amino acids present in the BSA peptide, enabling the identification and analysis of its structural components using Raman spectroscopy.

Furthermore, one of the remarkable features of the developed enhancing substrate is its reusability, as mentioned above. Even after the deposition of BSA on the substrate (Figure 6, blue line), the substrate can be effectively washed in an ethanol solution, resulting in the removal of BSA molecules and restoring the substrate to its original state (Figure 6, gray line). This exceptional stability and reusability make the enhancing substrate highly practical and cost-effective for multiple applications.

Finally, the enhanced Raman spectrum of BSA allows the identification of specific amino acids, even at the BSA concentration levels of 55 μg mL^−1^. The latter falls between the concentrations required for fluorescent methods (~5 mg mL^−1^, Ref. [38]) and specially designed ELISA (~5 ng mL^−1^, Ref. [39]).

## 4. Conclusions

To conclude, we showed a facile, cost-effective, and rapid route for preparing the enhancing Raman substrate based on gold nanoparticles with a mean diameter of 44 nm covered with a thin SiO_2_ shell (roughly 4 nm thick) in a space confined by Kapton tape using a drop-casting procedure. The characterized enhancing substrates exhibited a linear dependence between Raman intensity and the loading of MG dye, serving as a model molecule. This finding was further supported by Raman scattering mapping, which confirmed the uniform distribution of hotspots and Raman scattering intensity on the enhancing surface. The calculated enhancement factor of approximately 4 × 10^4^ and LOD of approximately 2.9 μM demonstrated the capability of detecting various natural molecules. While this enhancement factor is lower than the anticipated values for pure electromagnetic mechanisms (which can reach up to 10^6^), it is important to note that the present Raman substrate provides a robust and reusable scaffold for enhanced Raman spectroscopy. The combination of gold nanoparticles with a thin SiO_2_ shell offers an efficient platform for signal enhancement and molecular detection. Although such an EF is an order of magnitude lower than anticipated for pure electromagnetic mechanism (up to 10^6^), the present Raman substrate nevertheless provides a scaffold for reusable substrates.

The practical application of the substrates was demonstrated by detecting and analyzing BSA as a model peptide molecule at a concentration level of 55 μg mL^−1^ with improved peak resolution, allowing for the identification of specific amino acids.

Notably, the prepared enhancing substrates exhibit excellent reusability, which was demonstrated by successfully reusing the same substrate through a simple washing process with an ethanol solution. This remarkable feature allows for the repeated use of the substrate without compromising its performance or effectiveness.

Overall, this study introduces a novel approach for the preparation of highly efficient and reusable SERS substrates based on gold nanoparticles. These substrates hold great potential for a wide range of applications in the field of analytical chemistry and biosensing.

## Figures and Tables

**Table 1:** Summary of UV–vis and DLS characterization of synthesized gold nanoparticles by different methods. Calculations of the mean diameter and concentration were performed based on Refs. [15,30].

Sample	<d>/nm	C/Particles μL^−1^	<d>_DLS_/nm	<ζ>_DLS_/mV
Seed AuNPs	13	4 × 10^9^	17 ± 3	−39 ± 3
Seed-mediated grown AuNPs	28	4 × 10^8^	44 ± 10	−39 ± 2
Seed-mediated grown AuNPs with SiO_2_ shell	35	3 × 10^8^	52 ± 20	−43 ± 2

**Table 2:** Experimental Raman shifts for malachite green dye and the corresponding attribution of bands to the vibration of structural motives.

ν_633_^экс.^	ν_633_	Vibration Type, Attribution	Refs.
529	–	Scissoring of phenyl-C-phenyl	785 nm: [26,32]
797	796/798	Out-of-plane C-H (ring), wagging C-H of the benzene ring	532 nm: [33], 633 nm: [25,27], 785 nm: [26,32,34]
915	916	Out-of-plane C-H (ring), stretching of the benzene ring	532 nm: [33,35] 633 nm: [25,27] 785 nm: [26,32,34]
1172	1173/1172	In-plane C-H (ring), δ(C-H)	532 nm: [33,35] 633 nm: [25,27] 785 nm: [26,32,34]
1217	—	Rocking of C-H bonds, δ(C–H)	532 nm: [33] 633 nm: [2,26,34]
1368	1366/1365	Stretching of N-phenyl	532 nm: [33,35] 633 nm: [25,27] 785 nm: [26,34]
1395	1395	Stretching of N-phenyl, δ(C–H), Stretching of the benzene ring	532 nm: [33,35] 633 nm: [25] 785 nm: [26,32]
1616	1615/1613	Stretching of the benzene ring	532 nm: [33,35] 633 nm: [25,27] 785 nm: [26,32,34]

**Table 3:** Experimental Raman shifts for BSA peptide and the corresponding attribution of bands.

ν_633_^exp^	ν_633_	Vibration Type, Attribution	Refs.
620	621	Phenylalanine	[22]
640	642	Tyrosine	[22]
712	713	ν(C–S)	[3]
762	764	Tryptophan	[22]
833	833/827	Tyrosine/Tryptophan	[22,23]
859	856	Tyrosine	[23]
880	883	Tryptophan	[23]
1005	1003/1004	Phenylalanine	[3,22]
1045	1035	Phenylalanine	[22]
1170	1178	Tyrosine	[3]
1241	1252/1248	Various C-N (Amide III)	[3,22]
1315	1319	Histidine	[23]
1355	1365	Tryptophan	[23]
1412	1409	Lysine	[23]
1451	1444/1449	δ(CH3) in Methionine/Histidine/Lysine/Tryptophan	[3,22]
1545	1556	Tryptophan	[22]
——	1583/1587	Phenylalanine	[3,22]
1606	1609	Phenylalanine	[23]

## Data Availability

The data presented in this study are available on request from the corresponding author.
